# Transformer 2β regulates the alternative splicing of cell cycle regulatory genes to promote the malignant phenotype of ovarian cancer

**DOI:** 10.32604/or.2023.030166

**Published:** 2023-07-21

**Authors:** TING ZHOU, PEIYING FU, DONG CHEN, RONGHUA LIU

**Affiliations:** 1 Department of Obstetrics and Gynecology, Tongji Hospital, Tongji Medical College, Huazhong University of Science and Technology, Wuhan, China; 2 Center for Genome Analysis, ABLife BioBigData Institute, Wuhan, China

**Keywords:** TRA2B, CYR61, HMGA2, RNA binding proteins, Splicing, Ovarian cancer

## Abstract

Late-stage ovarian cancer (OC) has a poor prognosis and a high metastasis rate, but the underlying molecular mechanism is unclear. RNA binding proteins (RBPs) play important roles in posttranscriptional regulation in the contexts of neoplasia and tumor metastasis. In this study, we explored the molecular functions of a canonical RBP, Transformer 2β homolog (TRA2B), in cancer cells. TRA2B knockdown in HeLa cells and subsequent whole-transcriptome RNA sequencing (RNA-seq) analysis revealed the TRA2B-regulated alternative splicing (AS) profile. We disrupted TRA2B expression in epithelial OC cells and performed a series of experiments to confirm the resulting effects on OC cell proliferation, apoptosis and invasion. TRA2B-regulated AS was tightly associated with the mitotic cell cycle, apoptosis and several cancer pathways. Moreover, the expression of hundreds of genes was regulated by TRA2B, and these genes were enriched in the functions of cell proliferation, cell adhesion and angiogenesis, which are related to the malignant phenotype of OC. By integrating the alternatively spliced and differentially expressed genes, we found that AS events and gene expression were regulated independently. We then explored and validated the oncogenic functions of TRA2B by knocking down its expression in OC cells. The high TRA2B expression was associated with poor prognosis in patients with OC. In ovarian tissue, TRA2B expression showed a gradual increasing trend with increasing malignancy. We demonstrated the important roles of TRA2B in ovarian neoplasia and aggressive OC behaviors and identified the underlying molecular mechanisms, facilitating the targeted treatment of OC.

## Introduction

Ovarian cancer (OC) is the most common cause of gynecological cancer-associated death in postmenopausal women. Surgery and platinum-based cytotoxic chemotherapy are often futile for advanced OC and can cause physical deformation [1]. Therefore, early detection of OC is critical. Genetic mutations in key genes, including BRAF, KRAS, PTEN, P53 and BRCA1/2, are considered potential molecular drivers of OC [2]. As master regulators of posttranscriptional regulation, RNA binding proteins (RBPs) play crucial roles in RNA metabolism and regulatory RNA splicing, localization, monitoring, degradation and translation [3,4]. Since RBPs participate in the regulation of various splicing changes in cancers, they can contribute to several cancer hallmarks by affecting the expression patterns of important protein isoforms that regulate cell behavior and translational regulation [5,6]. However, the functions of RBPs in OC are largely unknown.

TRA2B, also called SFRS10 and SRFS10, is a member of the serine/arginine (S/R)-rich splicing factor (SRSF) family and was initially recognized as an important regulator of sex determination in insects [7–9]. The transformer 2α (TRA2A) gene was copied from the Transformer 2β homolog (TRA2B) gene early in the vertebrate lineage; therefore, these genes are conserved across the animal kingdom [10]. Serine phosphorylation is important for the activity and subnuclear localization of TRA2B [11,12]. As canonical RBPs, transformer proteins control the fate of target RNAs by regulating primary RNA alternative splicing (AS) and RNA degradation [13]. In addition to performing functions in sex determination, TRA2B is involved in other biological processes (BPs), including metabolism [14,15] and development [16,17]. A recent study demonstrated that Tra2α and Tra2β jointly control constitutive splicing and AS patterns via paralog compensation [18]. Collectively, these results suggest that TRA2B is a multifunctional protein and that its roles in the development of OC should be further explored.

Several studies have revealed that TRA2B can inhibit cellular apoptosis or promote invasion in several cancers [19,20]. Both the TRA2B messenger RNA (mRNA) and Tra2β protein levels are increased in many cancers [21]. The identified pro-oncogenic splicing targets of Tra2β include CD44 [22], HipK3 [23], and Nasp-T [24]. Increased protein and mRNA levels of S/R-rich proteins, including Tra2β and YB-1, have been observed in malignant OC tissues [25]. Moreover, a recent study reported that TRA2B is a proto-oncogene that may contribute to altering the expression of CYR61, which is related to cancer cell proliferation and apoptosis [26]. Thus, exploring the role of TRA2B in OC cells may greatly contribute to our knowledge of OC.

In this study, we used next-generation RNA sequencing (RNA-seq) to systematically investigate global TRA2B-regulated alternative splicing events (RASEs) and global gene expression changes after TRA2B silencing in cancer cells. We found that TRA2B extensively regulated both gene expression and alternative splicing events (ASEs) in HeLa cells and that these activities were highly related to its oncogenic functions. Silencing of TRA2B in OC cells validated its roles in the proliferation, invasion and apoptosis of OC cells. In summary, we have extensively confirmed that TRA2B exerts oncogenic functions in OC cells by regulating both the expression and AS of associated genes.

## Materials and Methods

### Cell culture and transfection

The human cervical carcinoma (CC) cell line HeLa (CCTCC® GDC0009) was obtained from the China Center for Type Culture Collection (Wuhan, Hubei, China). The human OC cell lines HO8910 and A2780 were obtained from the American Type Culture Collection (Rockville, MD, USA). HeLa, HO8910 and A2780 cells were cultured in Dulbecco’s modified Eagle’s medium (DMEM) supplemented with 10% fetal bovine serum (FBS), 100 µg/mL streptomycin, and 100 U/mL penicillin at 37°C in 5% CO_2_. Plasmid transfection of HeLa cells was performed using Lipofectamine 2000 (Invitrogen, Carlsbad, CA, USA) according to the manufacturer’s protocol. The transfected cells were harvested after 48 h for reverse transcription–quantitative polymerase chain reaction (RT–qPCR) analysis. The efficient TRA2B short hairpin RNA (shRNA) sequence was 5′-TACTCACCTCGTCGCTATTAA-3′. The small interfering RNA (siRNA) sequences were as follows: TRA2B siRNA 405 sense: 5′-GGUCUUACAGUCGAGAUUATT-3′, antisense: 5′-UAAUCUCGACUGUAAGACCTT-3′; TRA2B siRNA 581 sense: 5′-CCCAUUGCCGAUGUGUCUATT-3′, antisense: 5′-UAGACACAUCGGCAAUGGGTT-3′; TRA2B siRNA 798 sense: 5′-GCUCUCGCCGUCGGGAUUATT-3′, antisense: 5′-UAAUCCCGACGGCGAGAGCTT-3′; and siRNA negative control (NC) sense: 5′-UUCUCCGAACGUGUCACGUTT-3′, antisense: 5′-ACGUGACACGUUCGGAGAATT-3′.

### Assessment of shRNA knockdown effects

Glyceraldehyde-3-phosphate dehydrogenase (GAPDH) was used as the control gene to assess the effects of TRA2B knockdown. Complementary DNA (cDNA) synthesis was performed using standard procedures, and RT–qPCR was performed on a Bio-Rad S1000 instrument with Bestar SYBR Green RT–PCR Master Mix (DBI Bioscience, Shanghai, China). The level of each transcript was then normalized to the GAPDH mRNA level using the 2^−ΔΔCT^ method [27]. Data were compared with paired Student’s *t* test using GraphPad Prism software (San Diego, CA, USA). The sequences of the primers were as follows: TRA2B forward: 5′-TTATACCCGGTCACGGTCTC-3′, reverse: 5′-AGCTCAGCCCAAATACTCCA-3′; CYR61 forward: 5′-ACCCTCGGCTGGTCAAAGT-3′, reverse: 5′-GCTTCAGTGAGCTGCCTTTT-3′; CTGF forward: 5′-ACAGGCGGCTCTGCTTCTC-3′, reverse: 5′-GGCCCAGACCCAACTATGA-3′; FN1 forward: 5′-CACTCATCTCCAACGGCATAA-3′, reverse: 5′-GGCTTGAACCAACCTACGG-3′; and HMGA2 forward: 5′-CCTCTGCGACCTCAAAGCC-3′, reverse: 5′-CGCTGCCACCATCAACACC-3′.

### Cell proliferation assay

A 3-(4,5-dimethylthiazol-2-yl)-2,5-diphenyl-2H-tetrazolium bromide (MTT) assay was used to assess cell proliferation. In brief, HeLa cells were cultured in 96-well plates and then transfected with the vector using Lipofectamine 2000 (Invitrogen, USA) according to the manufacturer’s protocol. The cells were then incubated at 37°C for 48 h. Subsequently, MTT solution (5 mg/mL [0.025 mL]) was added to each well, and the cells were incubated for another 4 h. After centrifugation, the supernatant was removed from each well. The colored formazan crystals produced via the reduction of MTT in each well were dissolved in dimethyl sulfoxide (DMSO; 0.15 mL), and the optical density (OD) was measured at 490 nm. The cytotoxic effects of TRA2B shRNA transduction were determined using a Cell Counting Kit-8 (CCK-8) assay. Cells were cultured in 96-well plates at a density of 10^4^ cells/well and incubated overnight at 37°C with 5% CO_2_. TRA2B shRNA, TRA2B siRNA 405, TRA2B siRNA 581 and TRA2B siRNA 798 were added to triplicate wells of the 96-well plates, and the plates were incubated for 0, 12, 24, 48, or 72 h. Then, 10 µL of CCK-8 solution was added to each well, and the plates were incubated for 2 h at 37°C with 5% CO_2_ according to the manufacturer’s protocol (Dojindo, Japan). All *in vitro* experiments were repeated three times.

### Flow cytometric analysis of apoptosis

HeLa, HO8910 and A2780 cells were seeded in 24-well culture plates. At 70% confluence, the cells were transfected with the vector using Lipofectamine 2000 according to the manufacturer’s protocol. The cells were then incubated at 37°C for 48 h, and the viable cells were harvested and washed twice with phosphate-buffered saline (PBS). The viable cells were then double-stained with fluorescein isothiocyanate (FITC)-conjugated annexin V and 7-amino actinomycin D (7-AAD; Biotech Co., Ltd., Suzhou, Jiangsu, China). The apoptosis rate was defined as the sum of the number of cells in the lower right and upper right quadrants. The induction of apoptosis was detected using an annexin V/propidium iodide (PI) apoptosis detection kit (Nanjing Key Gen Biotech Co., Jiangsu, China) according to the manufacturer’s instructions. In brief, cells were cultured at a density of 1.0 × 10^5^ cells/mL, treated with TRA2B shRNA/TRA2B siRNA and cultured for 12 h at 37°C with 5% CO_2_. All samples were analyzed using a FACSort flow cytometer (Becton Dickinson, USA).

### Western blot (WB) analysis

Proteins were extracted from HO8910 and A2780 cells cultured for 30 min in a 37°C atmosphere containing 5% CO_2_. The extracted proteins were separated by sodium dodecyl sulfate‒polyacrylamide gel electrophoresis (SDS‒PAGE) and electrophoretically transferred onto nitrocellulose membranes (Millipore, USA). Standard WB analyses were performed using antibodies specific for CTGF (Santa Cruz, CA, USA), CYR61 (Santa Cruz, USA), FN1 (Sigma), HMGA2 (Sigma), ASAP3 (Sigma, USA), ERBB3 (Sigma, USA), IL-6 (Sigma, USA), IL-1 (Sigma, USA), JUN (Invitrogen, USA), MAP2K8 (Sigma, USA), MMP13 (Sigma, USA), NPNT (Invitrogen, USA), ODC1 (Invitrogen, USA), VEGFC (Sigma, USA) and GAPDH (Santa Cruz, USA). Immunoreactions were visualized using a horseradish peroxidase (HRP)-conjugated goat anti-rabbit secondary antibody (Santa Cruz, CA, USA) and detected by enhanced chemiluminescence. BandScan software was used to analyze the grayscale values.

### Wound healing migration assay

HO8910 and A2780 cells (2 × 10^5^) were seeded in 6-well culture plates. After the confluent monolayers were scraped, the plates were washed twice to remove detached cells, and the attached cells were incubated in growth medium. After 0 and 24 h, cell migration was assessed by imaging, and the area newly covered with cells was measured with ImageJ software.

### Transwell assay

To determine whether TRA2B can influence the invasiveness of OC cells, a Transwell assay was performed. The membrane in the upper chamber was coated with 100 µL of 1 mg/mL Matrigel (BD, USA). After the Matrigel solidified, 200 µL of the cell suspension was added to the upper Transwell chamber, and the cells were cultured for 24 h at 37°C. Then, the cells in the upper chamber were removed, and the cells on the lower surface of the membrane were fixed with 7% ice-cold ethanol and stained with 0.5% crystal violet for 30 min. The cells were counted with Image-Pro Plus.

### RNA extraction and sequencing

HeLa cells were ground into a fine powder before total RNA was extracted with TRIzol (Ambion, Shanghai, China). RNA was further purified by two rounds of phenol–chloroform extraction and was then treated with RQ1 DNase (Promega, Madison, WI, USA) to remove DNA. The quality and quantity of the purified RNA were determined by determining the 260 nm/280 nm absorbance ratio (A260/A280) using a SmartSpec Plus spectrophotometer (Bio-Rad, Hercules, California, USA). The integrity of the RNA was further verified by 1.5% agarose gel electrophoresis.

For each sample, 1 µg of total RNA was used for RNA-seq library preparation using a VAHTS Stranded mRNA-seq Library Prep Kit (Vazyme, Nanjing, Jiangsu, China). Polyadenylated mRNA was purified, fragmented and converted into double-stranded cDNA. After end repair and A-tailing, the DNA fragments were ligated to VAHTS RNA adaptors (Vazyme). The purified ligation products with lengths of 200–500 base pairs (bp) were digested with heat-labile uracil-DNA glycosylase (UDG), and the single-stranded cDNA was amplified, purified, quantified, and stored at −80°C before sequencing.

For high-throughput sequencing, the libraries were prepared in accordance with the manufacturer’s instructions and subjected to 150-nucleotide (nt) paired-end sequencing on an Illumina HiSeq X Ten system.

### Processing and alignment of raw RNA-seq data

Raw reads containing more than 2 N bases were first discarded. Then, the adaptors and low-quality bases were trimmed from the raw sequencing reads using FASTX-Toolkit (version 0.0.13). Short reads of less than 16 nt were also excluded. The clean reads were then aligned to the GRch38 genome with TopHat2 [28]; 4 mismatches were allowed. The uniquely mapped reads were used to count the gene reads and calculate the fragments per kilobase of transcript per million fragments mapped (FPKM) values [29].

### Analysis of differentially expressed genes (DEGs)

The R Bioconductor packages edgeR [30] and DESeq [31] were utilized to identify DEGs. A false discovery rate (FDR) of <0.05 and a fold change (FC) of >2 or <0.5 were set as the cutoff criteria for identifying DEGs.

### AS analysis

The ASEs and RASEs in the samples were defined and quantified using the ABLas pipeline as described previously [32,33]. In brief, ABLas was used to detect 10 types of ASEs based on splice junction reads: exon skipping (ES) events, alternative 5′ splice sites (A5SSs), alternative 3′ splice sites (A3SSs), intron retention (IR) events, mutually exclusive exons (MXEs), mutually exclusive 5′ UTRs (5pMXEs), mutually exclusive 3′ UTRs (3pMXEs), cassette exons, A3SS&ES events, and A5SS&ES events.

To assess RBP RASEs, Student’s *t* test was used to evaluate the significance of the differences in ASE ratios. Events with a p value cutoff corresponding to a false discovery rate (FDR) cutoff of 5% were considered RBP RASEs.

### Functional enrichment analysis

To determine the functional categories enriched with the DEGs, the enriched Gene Ontology (GO) terms and Kyoto Encyclopedia of Genes and Genomes (KEGG) pathways were identified using the KOBAS 2.0 server [34]. The hypergeometric test and the Benjamini–Hochberg method for controlling the FDR were used to define the enrichment of each term.

### Kaplan–Meier (KM) Plotter analysis

The prognostic significance of the mRNA expression of AS-regulated genes in OC was evaluated using KM Plotter (www.kmplot.com), an online database containing microarray gene expression data and survival information for 1,657 clinical OC patients [35].

### Histopathological and immunohistochemical (IHC) analyses

OC samples and nontumor tissues were collected from the Department of Obstetrics and Gynecology, Tongji Hospital, Tongji Medical College, Huazhong University of Science and Technology. After ovarian tumors or ovaries were surgically removed, the tissues were fixed with formalin and embedded in paraffin. All sections were confirmed by hematoxylin and eosin (H&E) staining. IHC analyses were performed with the streptavidin–peroxidase complex method. In brief, paraffin-embedded tissues were sliced into 5-µm-thick sections and dewaxed. The sections were rehydrated with xylene and a graded alcohol series. Then, 30% hydrogen peroxide was added for 10 min to block endogenous peroxidase activity [36]. The tissue slides were incubated with a rabbit anti-TRA2B primary antibody (Abcam, 1:200; Cambridge, USA) at 4°C overnight and were then incubated with secondary antibodies and streptavidin–peroxidase complex reagent. Each section was evaluated by two pathologists in a blinded manner. Positive TRA2B staining was observed in the nucleus. Images were acquired with an Olympus microscope (Olympus, Tokyo, Japan). Average optical density (AOD) values were used to determine the percentages of positive staining and were quantified with ImageJ (http://rsbweb.nih.gov/ij/).

### Statistical analysis

All experiments were repeated at least three times, and all values are presented as the means±standard deviations (SDs). Data were analyzed with the SPSS statistical software package for Windows (Version 22.0, SPSS, Inc., Chicago, IL, USA). The statistical significance of differences between groups was determined by one-way analysis of variance (ANOVA); *p* < 0.05 indicated a significant difference.

## Results

### TRA2B plays important roles in ovarian neoplasia

**Figure 1:**
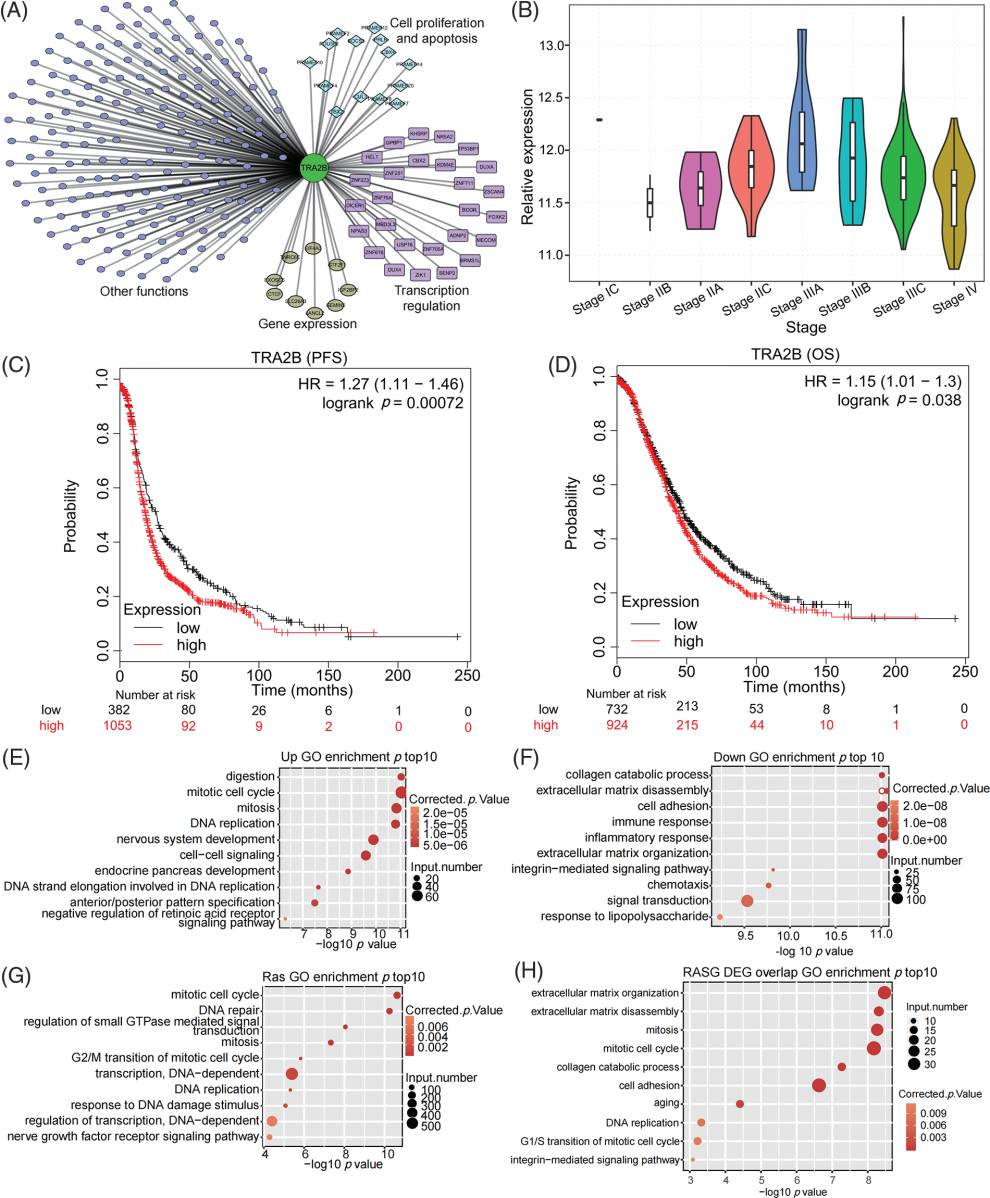
Analysis of transcriptome data in TCGA revealed the potential oncogenic function of TRA2B in OC. (A) Violin plot showing the expression pattern of TRA2B during the tumor stages of OC. Only one sample was obtained for stage IC. (B) Coexpression network of TRA2B and other genes. Functional clusters of coexpressed genes are labeled. (C, D) Survival (PFS and OS) analyses based on TRA2B in OC via KM Plotter demonstrated that TRA2B-high patients generally had shorter survival times than TRA2B-low patients. (E) Bubble plot showing the top 10 enriched GO BP terms enriched with the upregulated genes in TRA2B-high patients compared with TRA2B-low patients. (F) Bubble plot showing the top 10 GO BP terms enriched with the downregulated genes in TRA2B-high patients compared with TRA2B-low patients. (G) Bubble plot showing the top 10 GO BP terms enriched with RASGs between TRA2B-high and TRA2B-low patients. (H) Bubble plot showing the top 10 GO BP terms enriched with genes that were classified as both DEGs and RASGs between TRA2B-high and TRA2B-low patients.

As a splicing factor, TRA2B plays important roles in multiple BPs, but its functions and molecular mechanisms in OC are unclear. We constructed a TRA2B regulatory network by performing coexpression analysis (Pearson correlation coefficient ≥0.6 and *p* value ≤ 0.05). TRA2B was coexpressed with genes involved in cell proliferation/apoptosis, transcriptional regulation and gene expression (top enriched terms, Fig. 1A). By utilizing the transcriptome data of OC from The Cancer Genome Atlas (TCGA), we found that the TRA2B level first increased and then decreased as the OC stage increased (Fig. 1B), indicating the regulatory role of TRA2B in OC development. Analyses of the relationships of TRA2B expression with overall survival (OS) and progression-free survival (PFS) in OC via KM Plotter revealed that OC patients with higher TRA2B expression levels (TRA2B-high patients) generally had shorter survival times than patients with lower TRA2B expression levels (TRA2B-low patients) (*p* = 0.00072 for OS and *p* = 0.038 for PFS) (Figs. 1C and 1D). To further explore the molecular functions of TRA2B in OC, we selected the 20 OC samples with the highest TRA2B expression levels and 20 OC samples with the lowest TRA2B expression levels to identify TRA2B-regulated genes and RASEs. Analysis of the DEGs revealed 3231 and 2518 genes that were upregulated and downregulated by TRA2B, respectively. Functional enrichment analysis revealed that the terms mitotic cell cycle and DNA replication were significantly enriched with the upregulated genes (Fig. 1E), while the terms cell adhesion and immune/inflammatory response were significantly enriched with the downregulated genes (Fig. 1F), indicating the potential molecular functions of TRA2B in OC. We then analyzed TRA2B RASEs and the functions of the regulated alternative splicing genes (RASGs). Mitotic cell cycle-related terms were also enriched with the RASGs (Fig. 1G). The expression and AS patterns of 671 genes were affected by TRA2B. Functional terms, including mitotic cell cycle, DNA replication, cell adhesion, and extracellular matrix disassembly, were enriched with both the DEGs and the RASGs (Fig. 1H). Analysis of OC transcriptome data in TCGA indicated that TRA2B promotes OC development by regulating the cell cycle and cell adhesion at the transcriptional or posttranscriptional level.

### TRA2B-regulated genes are involved in multiple cancer-related functions

**Figure 2:**
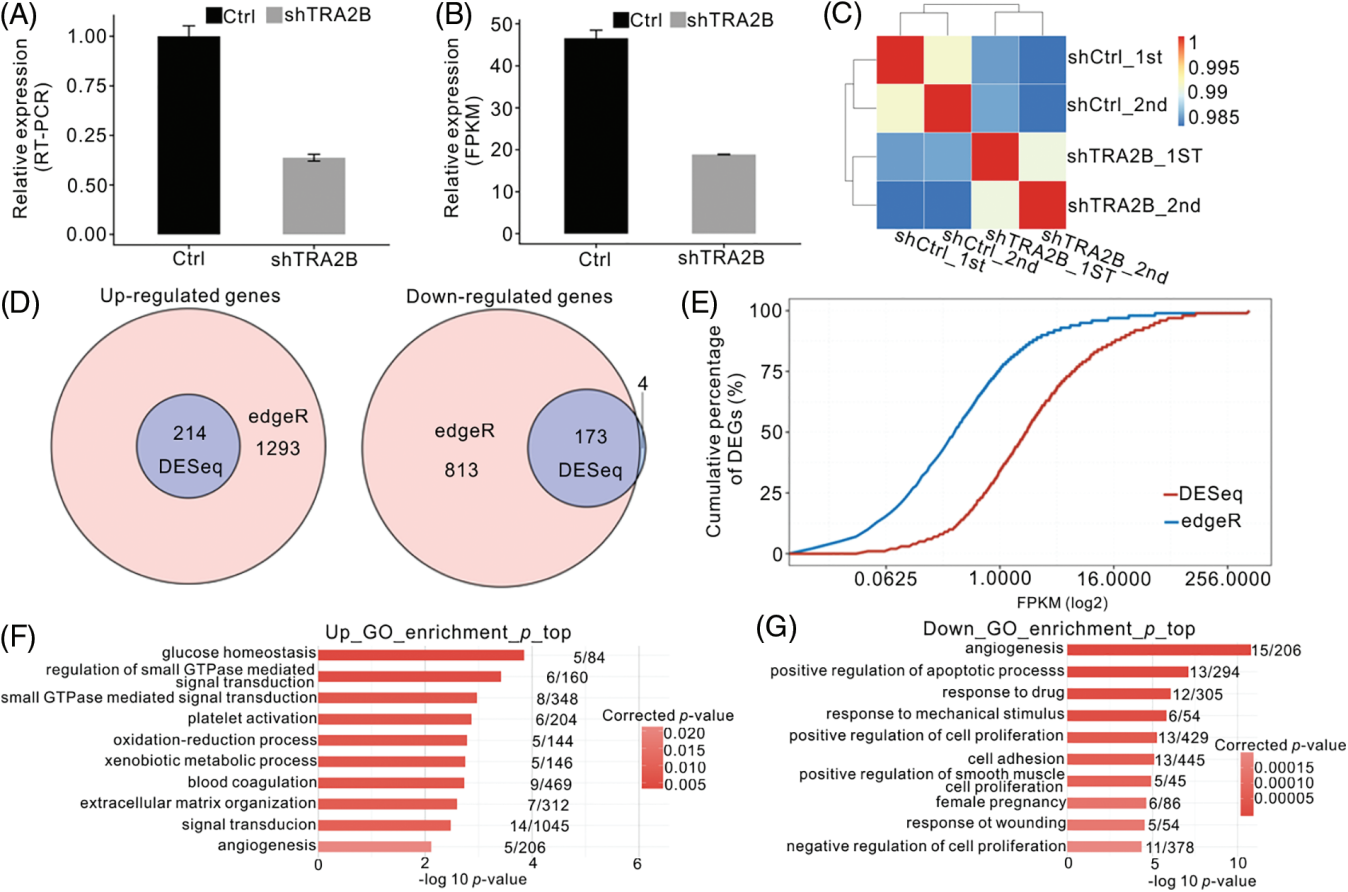
Global RNA-seq profile and DEGs regulated by TRA2B knockdown. (A, B) Bar plot of TRA2B mRNA expression levels in HeLa cells 48 h after transfection with a TRA2B-specific shRNA or a control vector, as measured by RT–qPCR (A), and FPKM values calculated by RNA-seq (B). (C) Heatmap showing the Pearson correlation matrix obtained by comparing the control vector- and TRA2B shRNA-treated samples. (D) Venn diagram showing the overlapping DEGs predicted by edgeR and DESeq. Upregulated (left panel) and downregulated (right panel) genes are shown. (E) Line plot showing the cumulative percentages of DEGs according to the FPKM values. (F and G) Bar plot showing the top ten GO BP terms enriched with the upregulated (E) and downregulated (F) genes.

To further elucidate the molecular mechanism of TRA2B-mediated transcriptional regulation, we constructed a cell model by knocking down TRA2B in HeLa cells using shRNAs. We examined the expression of TRA2B in HeLa cells transfected with two different empty vectors or shRNAs targeting TRA2B by RT–qPCR. We chose HeLa cells for these experiments because HeLa cells were used as model cells for studying RBPs in previous studies [37] and because HeLa cells are a good model for gene regulation studies involving cancer-related molecular mechanisms [38,39]. RT–qPCR analysis showed that 48 h after transfection, the TRA2B gene expression level in shTRA2B-transfected cells was 60% lower than that in control shRNA-transfected cells (Fig. 2A). We then constructed cDNA libraries of shTRA2B-transfected and control cells for RNA-seq analysis. Two biological replicates were prepared for the shTRA2B and control samples. After aligning the quality-filtered reads to the human GRCh38 genome sequence, the FPKM values were calculated and used as the expression levels of the identified genes. The effective silencing of TRA2B was also confirmed by parallel RNA-seq analysis (Fig. 2B). The gene expression levels were used to calculate a correlation matrix based on Pearson correlation coefficients. In the hierarchical clustering heatmap of sample correlations, the shTRA2B samples were clearly separated from the control samples, and the biological replicates were highly correlated (Fig. 2C), indicating that TRA2B knockdown was successful and altered the global expression profile.

To explore the impact of shTRA2B on gene expression in HeLa cells, we analyzed the DEGs between shTRA2B and control samples using the two most commonly used R packages, edgeR and DESeq. A FDR of <0.05 and a FC of >2 or <0.5 were used as the threshold criteria for identifying DEGs. edgeR predicted 1507 upregulated and 986 downregulated genes, and DESeq predicted 214 upregulated and 177 downregulated genes. Overlap analysis of the DEGs predicted by these two methods showed that except for 4 downregulated genes, all of the DEGs predicted by DESeq were also predicted by edgeR (Fig. 2D), indicating the high consistency between these two methods. However, the many additional DEGs predicted by edgeR did not meet the DEG criteria used by DESeq. By analyzing the cumulative expression curve of the DEGs, we found that the DEGs predicted by edgeR had significantly lower expression levels than the DEGs predicted by DESeq (Fig. 2E). To ensure the high credibility of our results, we used the DEG results obtained by DESeq in the following analysis.

We then determined the functions enriched with the DEGs by GO and KEGG enrichment analyses. Among the enriched GO BP terms associated with genes upregulated by shTRA2B, glucose homeostasis and small GTPase-mediated signal transduction were ranked at the top. The terms oxidation–reduction process and angiogenesis were also among the enriched BP terms (Fig. 2F). These metabolism and development terms are highly related to cancer development [40]. Angiogenesis, positive regulation of apoptotic process, and positive regulation of cell proliferation were the terms most highly enriched with the downregulated genes (Fig. 2G); these processes are also closely related to cancer hallmarks [41]. Together, these results suggest that TRA2B knockdown in HeLa cells substantially regulates the expression of cancer-related genes.

### TRA2B-regulated genes are involved in multiple cancer-related functions

**Figure 3:**
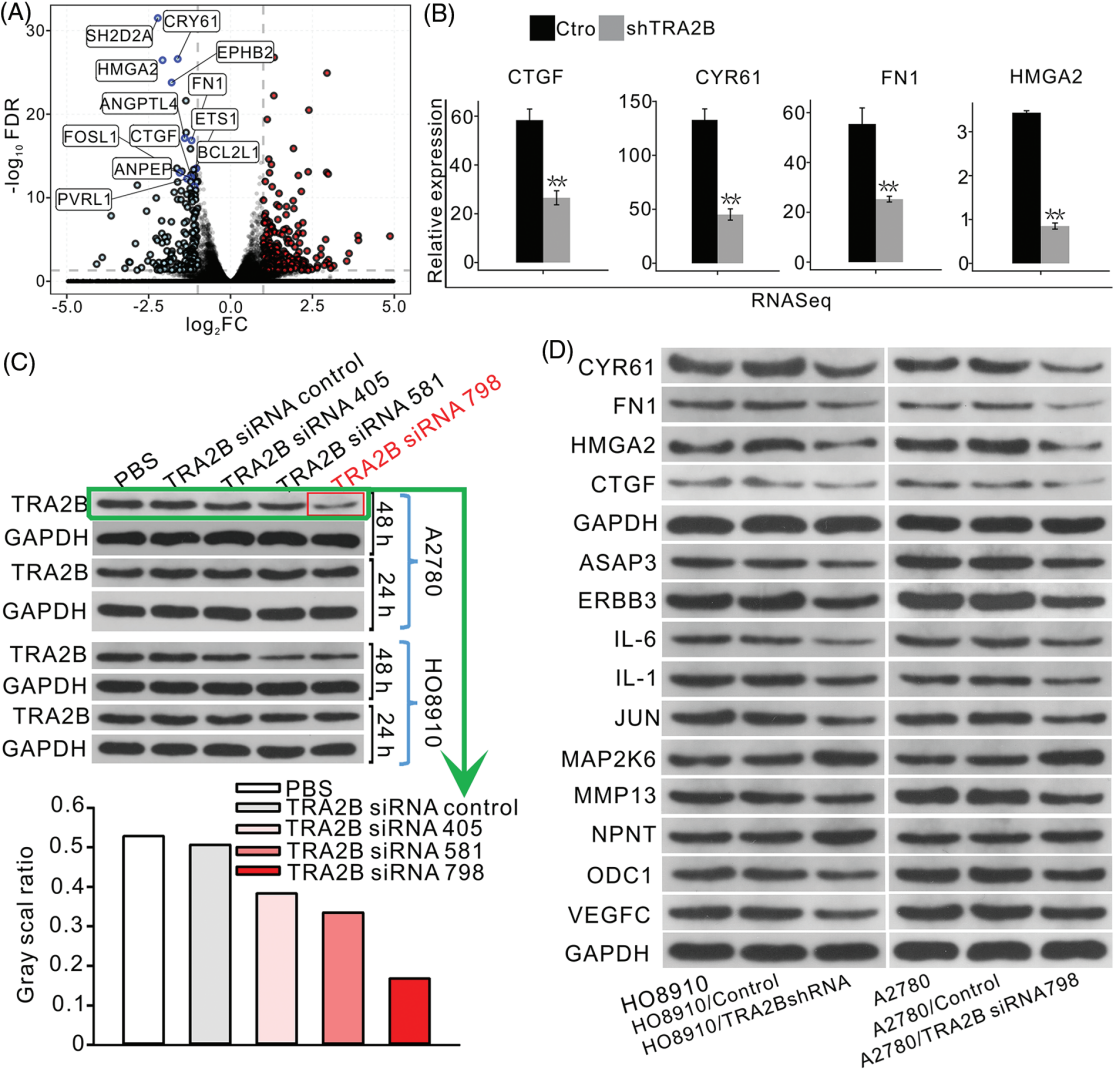
TRA2B significantly represses the expression of angiogenesis- and apoptosis-related genes. (A) Volcano plot showing the genes upregulated and downregulated by TRA2B knockdown. Genes with the top significant FDR values in the top 10 GO BP terms are shown in the figure. (B) Bar plot showing the differences in the expression of the selected genes between (A) control and TRA2B siRNA samples. (C) Screening and validation of the most effective TRA2B siRNA in OC cells. WB analysis of TRA2B expression in HO8910 and A2780 OC cell lines after siRNA treatment and the corresponding quantitative results. (D) WB analysis of selected proteins (CYR61, FN1, HMGA2, CTGF, ASAP3, ERBB3, IL-6, IL-1, JUN, MAP2K6, NPNT, ODC1 and VEGFC) regulated by TRA2B in OC cell lines and the corresponding quantitative results.

As indicated in Fig. 2G, the downregulated genes were associated with apoptosis and cell proliferation, which are directly related to the characteristics of cancer. The downregulated genes with the most significant FDR values among the top ten enriched GO BP terms, namely, CYR61, SH2D2A, HMGA2, EPHB2, FN1, ETS1, CTGF, ANGPTL4, FOSL1, BCL2L1, ANPEP, and PVRL1, are shown in a volcano plot (Fig. 3A). Only four genes were selected to verify the accuracy of the data, and the expression levels of these genes in OC cell lines were assessed to confirm that TRA2B regulates these genes. Other genes were also identified as DEGs. A list of the top 100 upregulated and top 100 downregulated genes is provided in the supplementary data (Suppl. Table S1). Individual analyses of the expression levels of several key genes showed that they were highly and consistently downregulated after TRA2B knockdown (Fig. 3B). Consistent with our findings, TRA2B was previously demonstrated to regulate the expression of CYR61 by directly binding to its pre-mRNA [26]. Three small siRNA fragments (siRNA 405, siRNA 581 and siRNA 798) were designed to screen for and validate the most effective TRA2B siRNA in OC cells, and siRNA 798 was found to exert the most obvious inhibitory effect on TRA2B expression in OC cells (Fig. 3C). To further validate the influence of TRA2B knockdown on the expression of these genes, we performed WB analysis to measure the corresponding protein levels in OC cells. After TRA2B knockdown, the expression of CYR61, FN1, HMGA2, CTGF, ASAP3, ERBB3, IL-6, IL-1, JUN, MMP13, ODC1, and VEGFC in OC cells was decreased. However, the expression of MAP2K6 and NPNT was increased (Fig. 3D). We used KM Plotter to analyze the survival statuses of OC patients with different expression levels of some key regulated genes (CYR61, FN1, HMGA2 and MAP2K6). OC patients with higher global CYR61, FN1 and HMGA2 expression levels had shorter survival times than patients with lower expression levels (Suppl. Figs. S1A–S1F). However, the trend for MAP2K6 was opposite that for the above genes (Suppl. Figs. S1G and S1H).

### Identification of TRA2B-dependent ASEs

**Figure 4:**
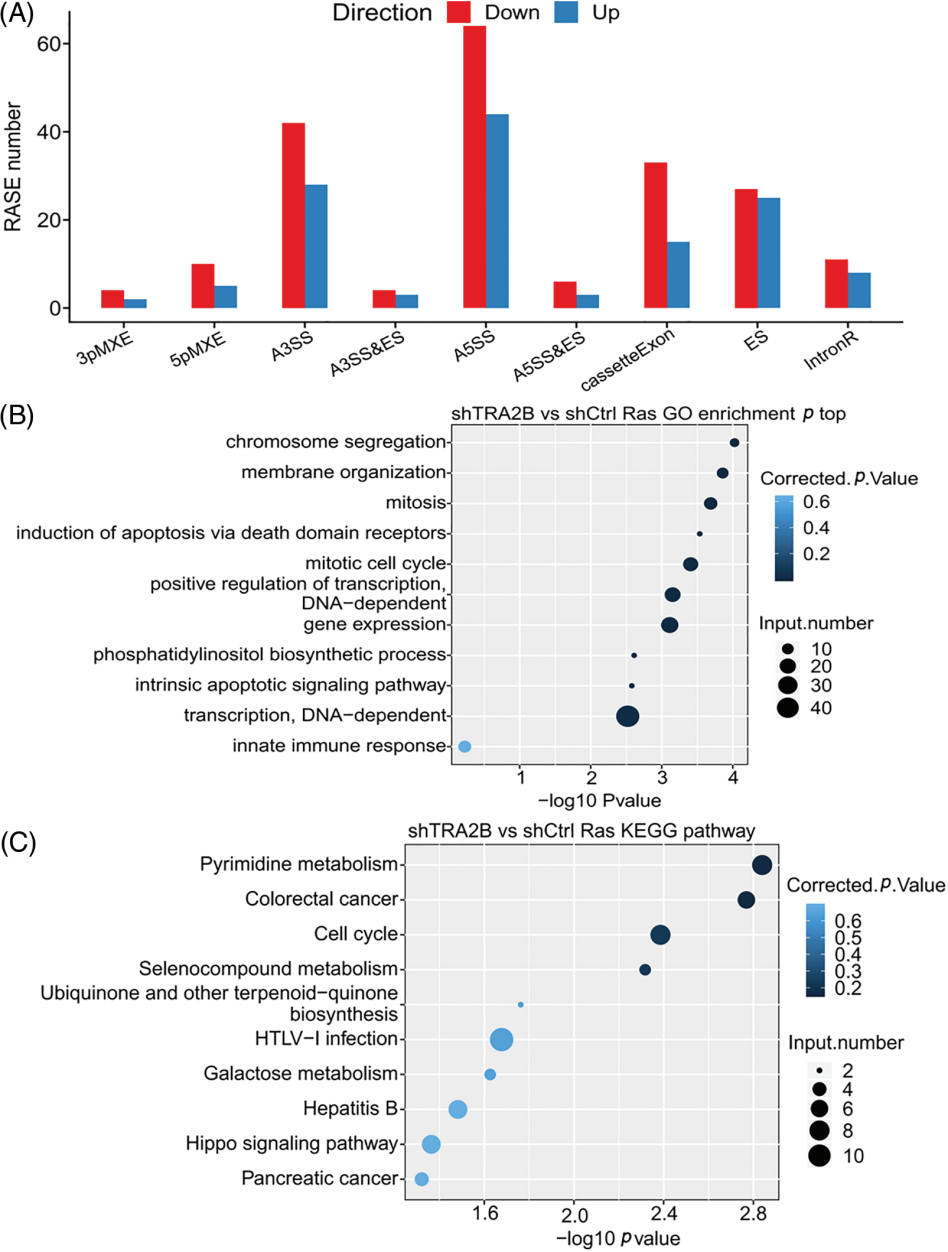
TRA2B regulates ASEs in HeLa cells. (A) Bar plot showing the number of RASEs in TRA2B knockdown compared with control cells. The RASEs are grouped by AS type. (B) Bar plot showing the top ten GO BP terms enriched with genes whose AS was regulated by TRA2B. (C) Bar plot showing the top ten KEGG pathways enriched with genes whose AS was regulated by TRA2B.

A key aim of this study was to further understand the regulatory effect of TRA2B on AS. Therefore, transcriptome sequencing data were further analyzed to investigate the RASEs upon TRA2B knockdown. We used the ABLas program [42] to detect RASEs influenced by TRA2B in HeLa cells. With an AS ratio of >0.15 and a *p* value of <0.05 as the threshold criteria, we identified 201 upregulated and 253 downregulated RASEs. The RASEs included cassette exon (69)/ES (86) events, A5SSs (127), and A3SSs (137). These data suggest that TRA2B globally regulates ASEs in HeLa cells. The other event types included 5pMXEs (n = 36), 3pMXEs (n = 17), MXEs (n = 29), A5SS&ES events (n = 12) and A3SS&ES events (n = 10) (Fig. 4A). These data indicate that TRA2B plays a role in the regulation of global ASEs in HeLa cells.

Further, the genes regulated by TRA2B-mediated AS were highly enriched in the GO BP terms chromosome segregation; mitosis; mitotic cell cycle; induction of apoptosis via death domain receptors; positive regulation of transcription, DNA-dependent; and gene expression (Fig. 4B). The enriched KEGG pathways (*p* < 0.05) included those involved in pyrimidine metabolism, colorectal cancer, the cell cycle, and selenocompound metabolism (Fig. 4C). In contrast to the functional enrichment of the DEGs, we found that the genes with RASEs were enriched mainly in cell cycle-related terms. These results were highly consistent with the TCGA data. The genes involved in the mitotic cell cycle included DSN1, MCM8, NSL1, CENPM, CDC16, CENPK, SKP1, SEC13, ANAPC11, UBA52, NEK9, NUP153, CLIP1, POLD4, CDK5RAP2, ESPL1, and RANBP2. Transcriptional regulation-related GO BP terms were also found to be enriched with genes whose AS was regulated by TRA2B, including SMAD4, SMAD2, HNRNPC, and HNRNPF.

**Figure 5:**
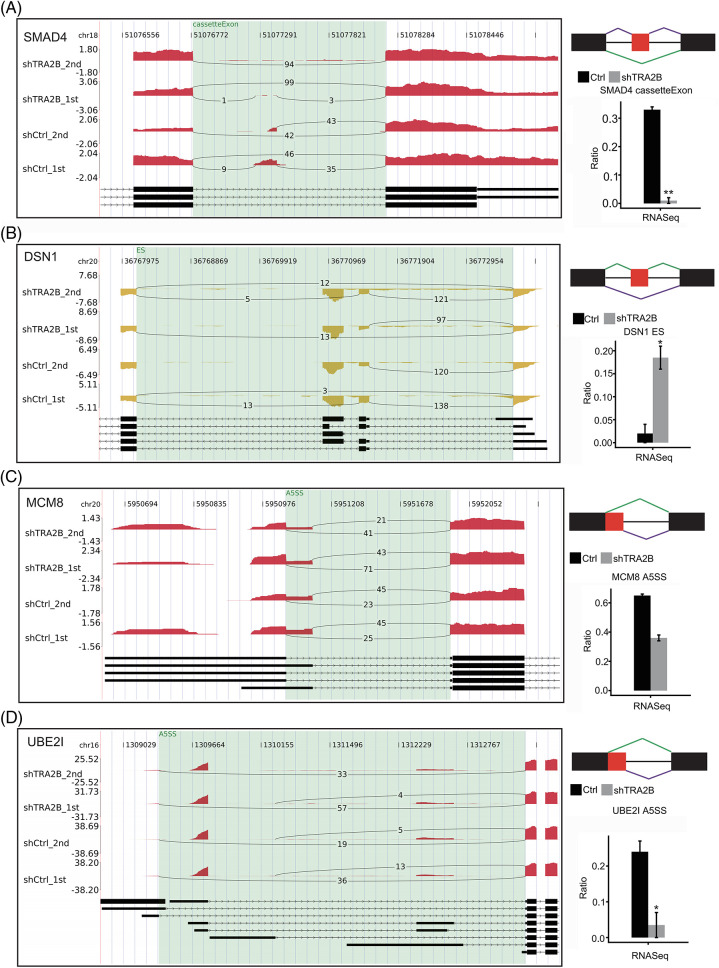
Presentation of TRA2B-affected RASEs. Three genes with RASEs, namely, SMAD4 (A), DSN1 (B), MCM8 (C) and UBE2I (D), are shown. Integrated Genomics Viewer (IGV) Sashimi plots showing the differences in AS between control and shTRA2B samples (left panel). The schematic diagrams show the structures of the ASEs (right panel, top) AS1 (shown in purple) and AS2 (shown in green); exon sequences are denoted by boxes, and intron sequences are denoted by horizontal lines. The quantitative RNA-seq results and statistical significance of the RASEs are shown (bottom right panels). The alteration rates of the RASEs were calculated using the formula AS1 junction reads/AS1 junction reads + AS2 junction reads.

To confirm the reliability of the RASE analysis results, we selected four RASEs and the corresponding genes, namely, SMAD4 (cassette exon, Fig. 5A), DSN1 (ES, Fig. 5B), MCM8 (A5SS, Fig. 5C), and UBE2I (A5SS, Fig. 5D), and determined their read densities and statistical significance. These four genes were related to the terms mitotic cell cycle and regulation of transcription, which were affected by TRA2B knockdown. In summary, these results suggest that TRA2B extensively regulates ASEs in cancer cells.

### TRA2B independently regulates the expression and AS patterns of genes

**Figure 6:**
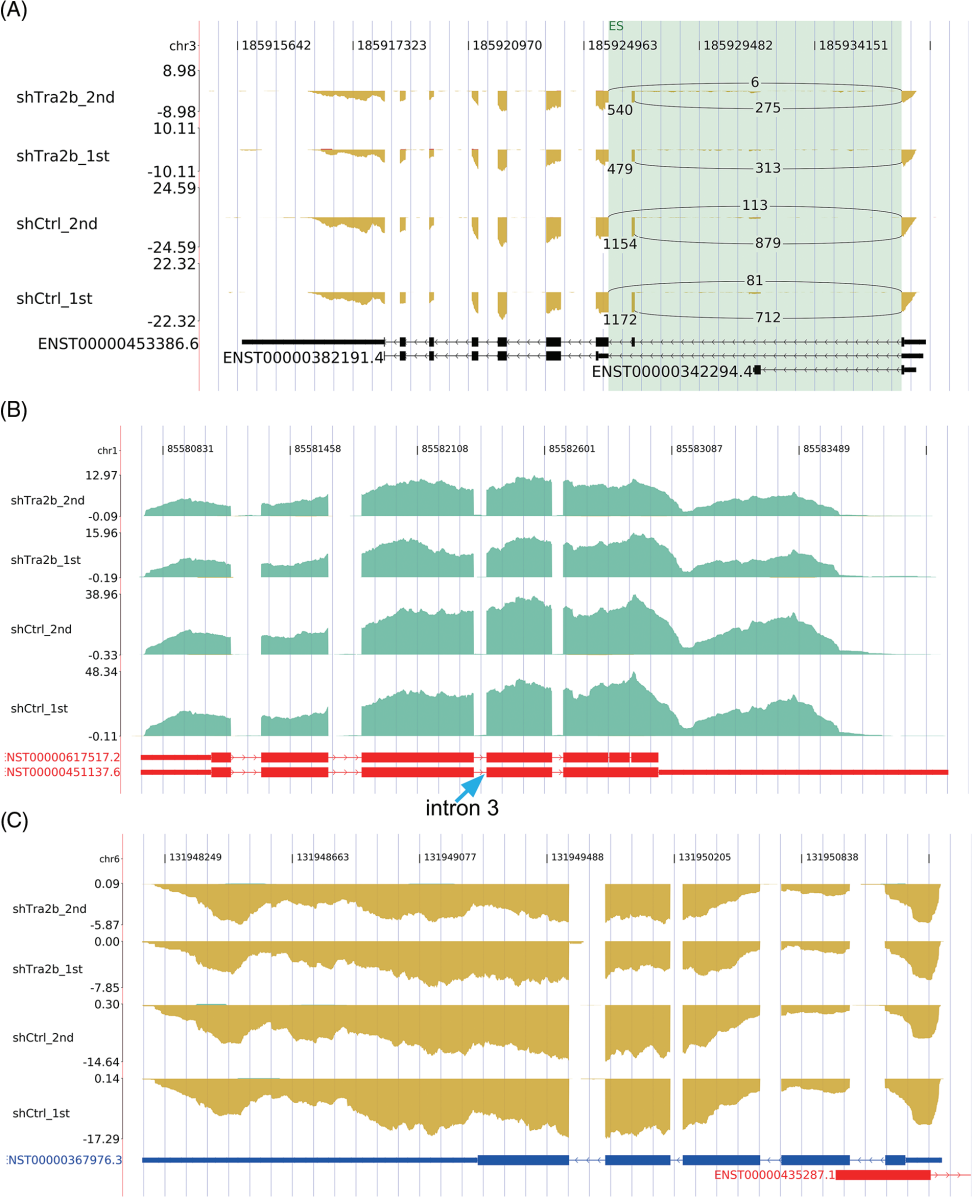
Analysis of the overlap between the DEGs and RASGs in TRA2B knockdown compared with control cells. (A) IGV Sashimi plots showing the changes in the AS and expression levels of the TRA2B gene in control and shTRA2B samples. (B) IGV Sashimi plots showing the changes in the AS and expression levels of the CYR61 gene in control and shTRA2B samples. (C) IGV Sashimi plots showing the changes in the AS and expression levels of the CTGF gene in control and shTRA2B samples.

To rule out the possibility that the increase in ASEs was simply due to upregulation of transcription, we identified overlapping genes whose expression levels and AS were both regulated by TRA2B knockdown. Six genes (Suppl. Fig. S2, *p* value = 1, hypergeometric test), namely, VIPR2, EPHB2, TRA2B, TSC22D3, NPNT and LINC00963, were identified. Interestingly, TRA2B knockdown promoted exon 2 inclusion in the TRA2B transcript (Fig. 6A). The small amount of overlap between the DEGs and RASGs with TRA2B knockdown suggested that TRA2B regulates the expression and AS patterns of genes via independent functional mechanisms.

To further validate our hypothesis, we constructed the read density profiles of two CCN family proteins (CYR61 and CTGF). CYR61 and CTGF play important roles in tumor progression and promote neovascularization and metastasis [42,43]. A previous study demonstrated that TRA2B binds to CYR61 RNA in exon 3 and retains intron 3 of CYR61 to regulate CYR61 protein expression [27]. Our results revealed no RASEs in the intron 3 region of TRA2B in either the control or shTRA2B samples (Fig. 6B, blue arrow). Similarly, no RASEs were detected in CTGF transcripts in the read density plot (Fig. 6C).

### TRA2B promotes the proliferation and invasion and inhibits the apoptosis of OC cells

**Figure 7:**
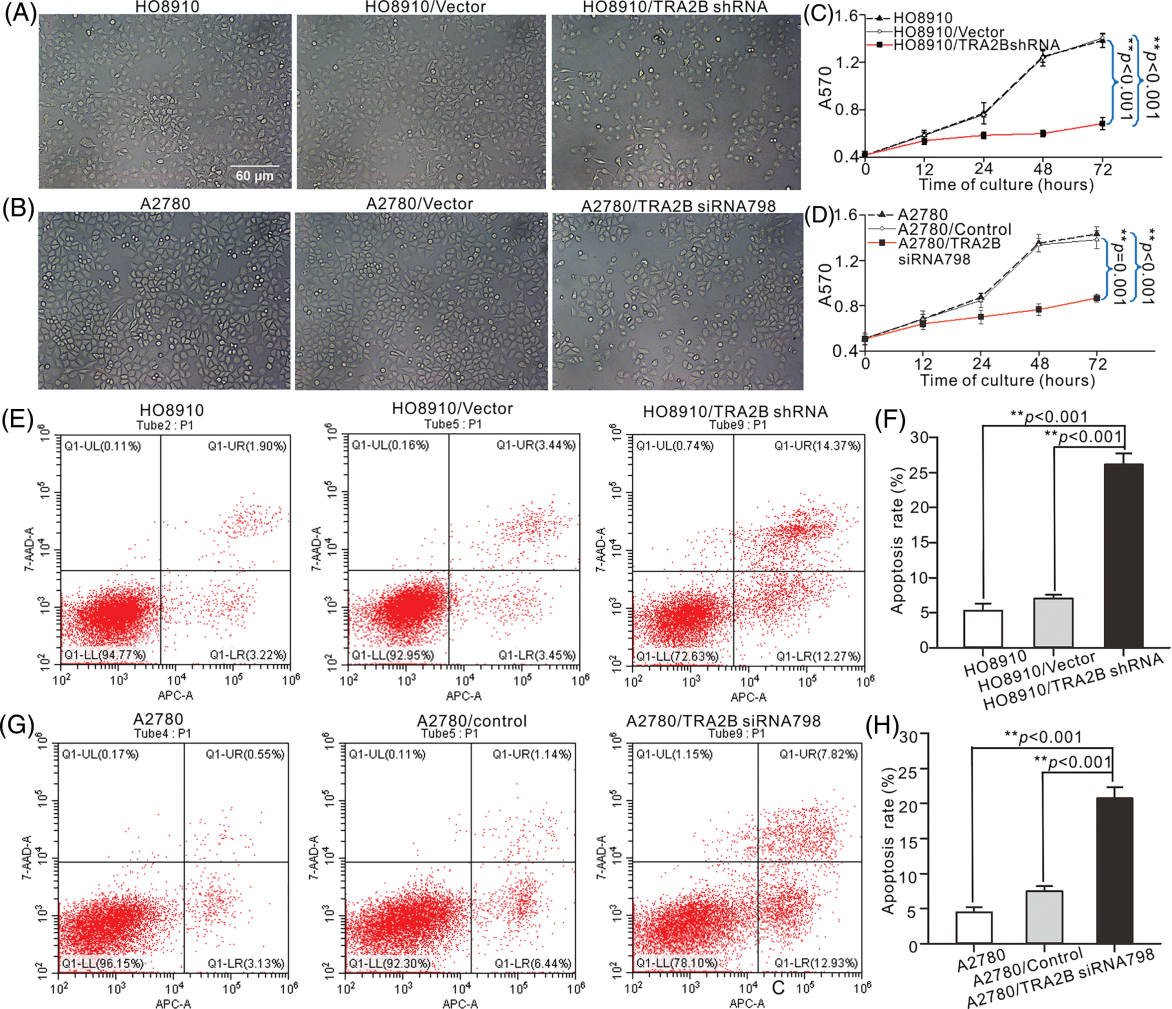
Effects of TRA2B knockdown on the proliferation and apoptosis of OC cells. The apoptosis rates of HO8910 and A2780 cells were determined using an annexin V/PI apoptosis assay. HO8910 and A2780 cell viability was assessed by microscopy after incubation with TRA2B siRNA 798, control or PBS for 48 h (A, HO8910; C, A2780). Cytotoxicity was quantified at different time points (0, 12, 24, 48, and 72 h) by a CCK-8 assay. The standard errors were calculated from three experiments and plotted (B, D). (E) HO8910 cells were incubated with TRA2B shRNA, vector, or PBS for 0, 12, 24, 48 and 72 h. Cell viability was assessed by microscopy. (G) A2780 cells were incubated with TRA2B siRNA 798, control or PBS for 0, 12, 24, 48 and 72 h. Scale bars, 100 µm. The standard errors were calculated and plotted (F, H). One-way ANOVA was performed to assess statistical significance: *p < 0.05; **p < 0.01.

To determine whether TRA2B affects cell proliferation, we observed morphological changes in various OC cell lines after treatment with TRA2B shRNA, TRA2B siRNA 798, vector/control or PBS. Most HO8910 and A2780 cells exposed to TRA2B shRNA or TRA2B siRNA 798 became rounded and detached from the tissue culture plate. Minor morphological changes were observed in cells treated with the vector/control for 48 h compared with control cells (Figs. 7A and 7B). Then, we used a CCK-8 assay to verify OC cell viability after treatment with TRA2B shRNA or TRA2B siRNA 798 for 0 h, 12 h, 24 h, 48 h and 72 h. TRA2B shRNA and TRA2B siRNA 798 had obvious cytotoxic effects on HO8910 and A2780 cells, respectively (Figs. 7C and 7D; right panels), and these effects were correlated with the morphological changes induced by TRA2B knockdown. TRA2B shRNA in HO8910 cells and TRA2B siRNA 798 in A2780 cells inhibited proliferation in a time-dependent manner. In addition, to determine whether TRA2B shRNA or TRA2B siRNA 798 induces apoptosis, the apoptosis rates of HO8910 and A2780 cells were analyzed using an annexin V/PI apoptosis assay. The apoptosis rates of TRA2B shRNA-treated HO8910 and TRA2B siRNA 798-treated A2780 cells were markedly higher than those of the corresponding vector/control-treated cells (Figs. 7E–7H).

**Figure 8:**
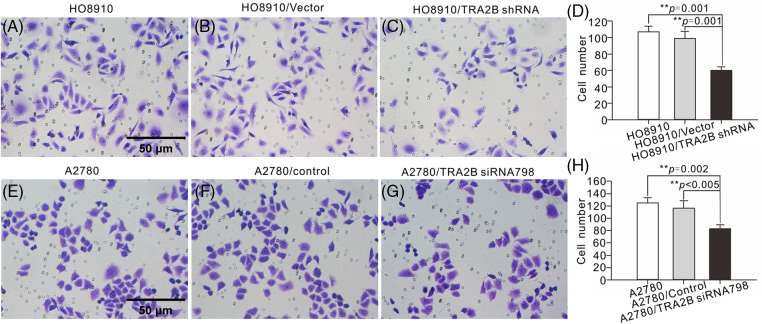
Effects of TRA2B knockdown on the migration and invasion of OC cells. Migration and invasion assays of HO8910 and A2780 cells were performed. (A–C) HO8910 cells were incubated with TRA2B shRNA, vector, or PBS for 48 h. (E–G) A2780 cells were incubated with TRA2B siRNA 798, control or PBS for 48 h. The standard errors were calculated and plotted (D, H) (right panels).

We next investigated whether TRA2B shRNA or TRA2B siRNA 798 could inhibit the migration and invasion of OC cells by conducting Transwell and wound healing assays. As the Transwell assay results showed, TRA2B shRNA and TRA2B siRNA 798 inhibited the invasion of OC cells (Fig. 8). Moreover, the wound healing assay results showed that downregulation of TRA2B mediated by TRA2B shRNA or TRA2B siRNA 798 significantly inhibited the migration of OC cells (Suppl. Fig. S3).

### TRA2B expression in different tissues

**Figure 9:**
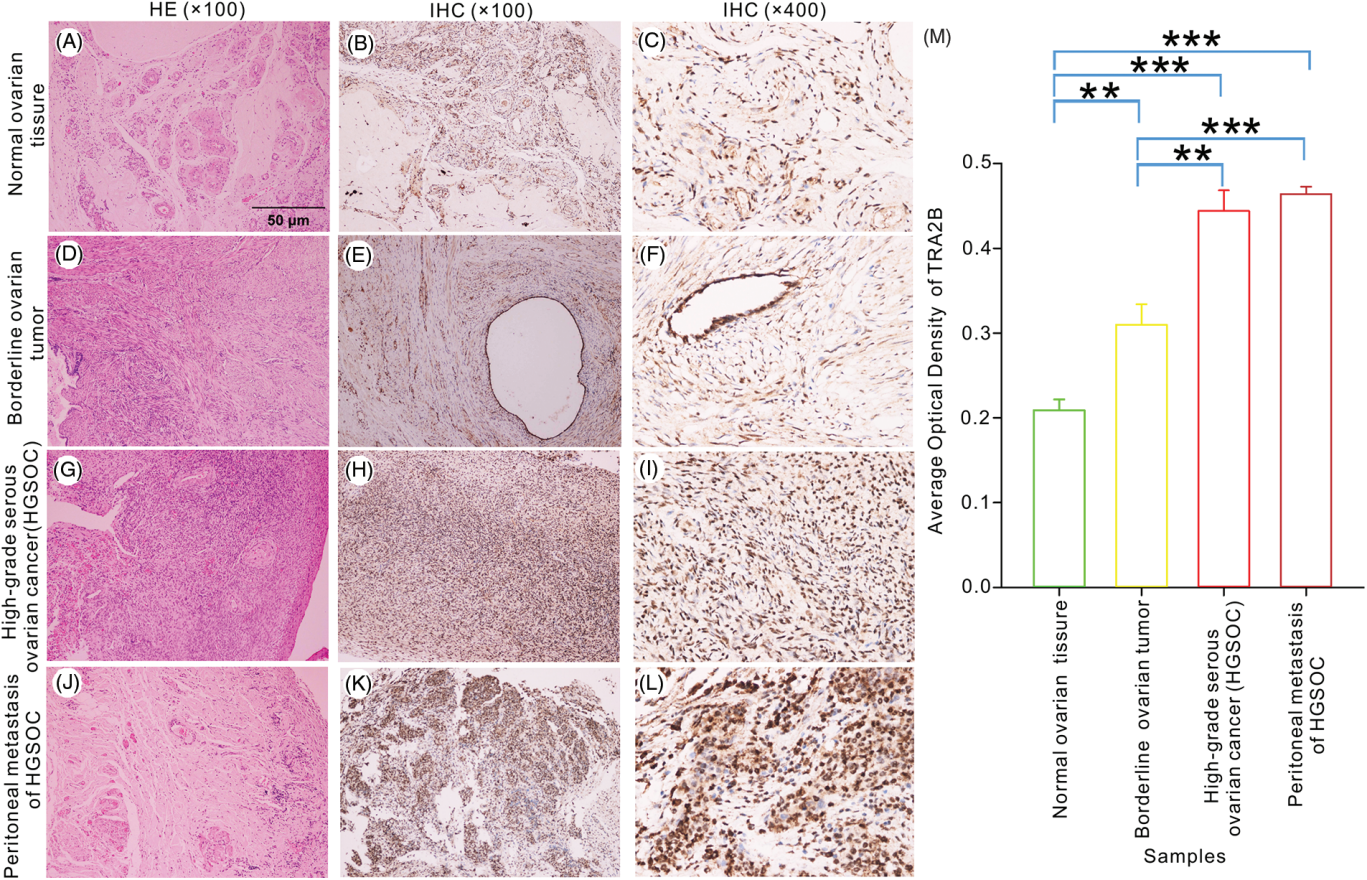
TRA2B expression in benign, peritumoral and malignant ovarian tumor tissues. IHC staining for TRA2B in nontumor ovarian tissue (B, C), borderline ovarian tumor tissues (E, F), HGSOC tissue (H, I) and peritoneal metastatic HSGOC tissue (K, L) (original magnification, 100× and 400×). H&E staining (A, D, G, J) (original magnification, 100×). The AOD was used to quantify TRA2B staining (M). All experiments were repeated three times. The data are presented as the means ± SDs: **p < 0.01 and ***p < 0.001.

To further investigate whether TRA2B maintains neoplastic properties of human ovarian tumors, serial tissue sections were stained with H&E to verify the histology of primary tumor tissues (Fig. 9, left panels). We assessed TRA2B expression in benign tissues (Fig. 9A–9C), borderline ovarian tumor tissues (Fig. 9D–9F), high-grade serous ovarian carcinoma (HSGOC) tissues (Fig. 9G–9I) and peritoneal metastatic tissue (Fig. 9J–9L). As shown in Fig. 9 the TRA2B expression levels were significantly higher in OC tissues and peritoneal metastatic HSGOC tissues than in benign tissues (*p* < 0.001). Furthermore, significant differences in the expression levels of TRA2B were observed between benign ovarian tissues and peritumoral tissues, and the differences in the TRA2B level between peritumoral samples and HSGOC samples were similar (*p* < 0.01). However, no difference in the expression of TRA2B was observed between primary tumor lesions and peritoneal metastases (Fig. 9M) (*p* = 0.252).

## Discussion

In this study, we used a high-throughput transcriptome sequencing approach to explore the influence of TRA2B on global gene expression and AS in human cancer cells and validated the cancer-promoting functions of TRA2B in OC cells. Relatively high concentrations of hyperphosphorylated TRA2B protein isoforms with altered expression have been detected in OC, suggesting that TRA2B is responsible for the known changes in AS in OC [44]. We also highlighted the functions of TRA2B in regulating expression in addition to its functions in regulating AS and found that these two regulatory functions may be independent of each other. Silencing of TRA2B expression in OC cells inhibited their migration and proliferation, whereas apoptosis was induced in these cells, confirming the oncogenic functions of TRA2B. Taken together, these results greatly expand the knowledge on the functions and molecular mechanisms of TRA2B in cancer.

The regulation of AS is a major mechanism underlying the enhancement of transcriptome and proteome diversity, particularly in mammals [45]. A large proportion of RBPs cooperate to control this process, and RNAs are ultimately processed into different mature RNA isoforms. Aberrant expression of RBPs can greatly alter the AS profiles in cells and lead to disease or dysplasia [46,47]. The canonical splicing factor TRA2B performs recognized AS-regulating functions in organisms from insects to mammals. TRA2B promotes HIV-1 RNA processing by directly binding to target RNAs [48,49]. Elevated expression levels of TRA2B have been observed in several cancers, including lung, ovarian, cervical, stomach, head, and neck cancers, in which these elevated levels are associated with neoplasia and metastasis [50]. To determine the underlying mechanisms, we profiled TRA2B RASEs. HeLa cells are the most widely used model cells worldwide and were therefore initially used herein to identify target genes regulated by TRA2B. Based on the results regarding the TRA2B RASEs in OC transcriptome data from TCGA, TRA2B has the potential to regulate the cell cycle by influencing the AS patterns of related genes, a finding that enhances the knowledge of the molecular mechanisms by which TRA2B participates in tumor development. We also found that TRA2B repressed the expression of genes associated with cell adhesion, suggesting that TRA2B contributes to cancer cell metastasis by changing the extracellular matrix composition. We validated the AS-regulating functions of TRA2B in OC patients and cancer cells. We discovered that mitotic cell cycle-related genes were significantly enriched among the TRA2B RASGs, providing an explanation for how TRA2B promotes cancer cell proliferation. Previous studies have suggested that TRA2B regulates the cell cycle by controlling a cassette exon in the NASP gene [22,25]. We identified several other genes controlling the cell cycle whose AS was regulated by TRA2B, including SMAD4, DSN1, MCM8, and UBE2I. Interestingly, TRA2B knockdown also significantly influenced the AS pattern of TRA2B itself (Fig. 6A), implying the existence of a regulatory feedback loop involving TRA2B and its RNA targets in cancer cells. Together, these results demonstrate the novel functional pathways through which TRA2B regulates the cell cycle in cancer cells.

Although RBPs primarily bind to RNAs to regulate their AS or stability, they also extensively interact with DNA to regulate gene expression [51]. Therefore, we next analyzed the regulatory effects of changes in TRA2B expression in HeLa cells and identified approximately four hundred DEGs between TRA2B knockdown and control cells. Functional analysis of these DEGs demonstrated their close relationships with cancer development. Most studies on TRA2B-mediated expression regulation have identified genes associated with the AS-regulating functions of TRA2B. Thus, we hypothesized that many DEGs are also regulated by AS. However, we found that TRA2B regulated both the expression and AS of only six genes. These results strongly suggest that TRA2B regulates gene expression via other important and undiscovered mechanisms. TRA2B has been reported to regulate gene expression by competing with RNA molecules or proteins [52] and can indirectly regulate gene expression by affecting the AS of genes with functions in transcriptional regulation. In this study, we found that the AS of several genes, including some genes encoding transcription factors (TFs), was regulated by TRA2B. Abnormal transcripts of these genes may encode nonfunctional proteins that cannot correctly regulate the transcription of downstream genes. In our study, the very small number of overlapping DEGs and RASGs supported our hypothesis that TRA2B-mediated regulation of gene expression and TRA2B-mediated regulation of AS do not occur simultaneously for the same gene. A regulatory axis connecting AS and gene expression may exist and needs further investigation.

The intensity of TRA2B expression in nontumor, peritumoral and HSGOC samples gradually increased with increasing malignancy. Thus, these data support the idea that TRA2B expression is involved in the progression of ovarian neoplasia. Using the OC transcription data in TCGA, we found that the expression level of TRA2B differed depending on the OC stage. Furthermore, the prognostic significance of TRA2B in OC patients was investigated using the KM Plotter online database, and the results suggested that high TRA2B expression is associated with poor patient survival. Therefore, TRA2B may play an important role in ovarian tumorigenesis. The detailed mechanism underlying this role merits further investigation.

A series of experiments performed herein confirmed that the proliferation, apoptosis and invasion of epithelial OC cells are significantly affected by disruption of TRA2B expression in tumor cells. Experiments on two different cancer cell lines confirmed that TRA2B indeed regulated target gene expression and that this finding was not an incidental phenomenon in cancer cells. We admit that some findings appear inconsistent, but this inconsistency does not affect the main conclusions of this study. On the other hand, analysis of transcriptome data In TCGA revealed that TRA2B plays an important regulatory role and performs an oncogenic function in OC development. Survival analysis showed that the OS times of patients were correlated with the expression levels of genes that were regulated by TRA2B in OC cells. Therefore, abnormal regulation of AS by TRA2B plays a vital role in the development of OC. The abnormal expression of TRA2B was also preliminarily confirmed in clinical OC specimens. However, this study has some limitations. Experiments with orthotopic mouse models of OC are currently being performed to further verify these findings and confirm that TRA2B functions as an RBP to affect AS in OC pathogenesis and to determine the clinical significance of TRA2B as a drug target.

## Supplementary Materials

FIGURE S1 Survival curves showing the OS times of patients with OC stratified into two subgroups according to the expression levels of CYR61, HN1, HMGA2 and MAP2K6. Survival analysis of patients with OC based on CYR61, HN1 and HMGA2 expression by KM plotter demonstrated that OC patients with high global expression levels of any these genes except for MAP2K6 had shorter survival times than patients with low expression levels.

FIGURE S2 Analysis of the overlap between DEGs and RASGs in TRA2B knockdown compared with control cells. Venn diagram showing the overlap between the DEGs and RASGs.

FIGURE S3 Downregulation of TRA2B inhibited the migration of OC cells. A wound healing assay was performed to investigate the migration of OC cells transfected with TRA2B shRNA or TRA2B siRNA 798 for 24 h (**A**, HO8910; **B**, A2780).

Table S1 The top 100 upregulated and downregulated genes

## Data Availability

The RNA-seq data associated with this publication have been deposited in the NCBI Gene Expression Omnibus and are accessible under GEO series accession number GSE176214.
