# Supplementary figures and images for: Transformer 2β regulates the alternative splicing of cell cycle regulatory genes to promote the malignant phenotype of ovarian cancer
Source: Oncol Res. 2023 Jul 21;31(5):769–85. doi: 10.32604/or.2023.030166 (PMC10398401; doi:10.32604/or.2023.030166)

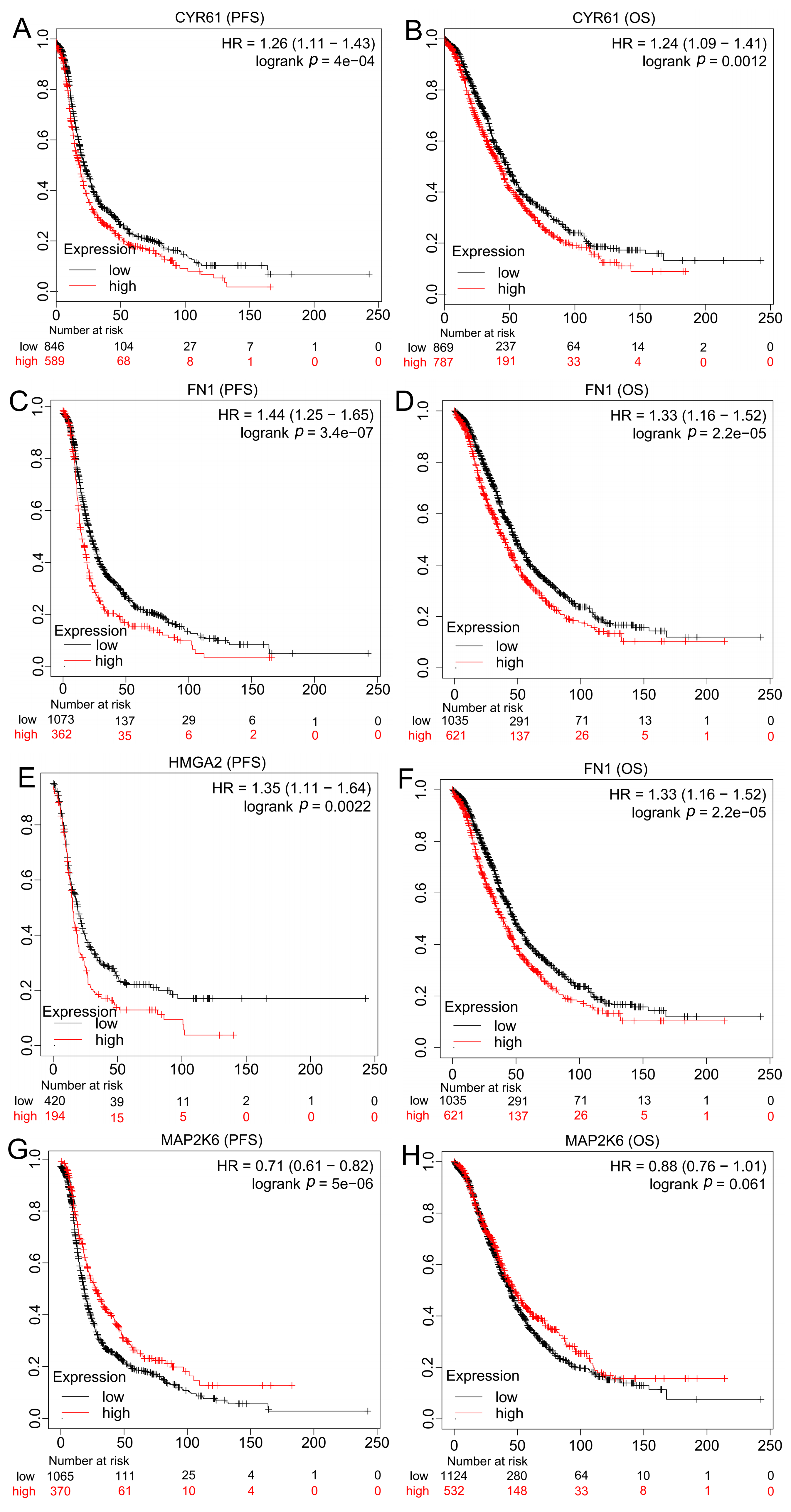

Supplement: FIGURE S1 [file OncolRes-31-30166-s001.tif]

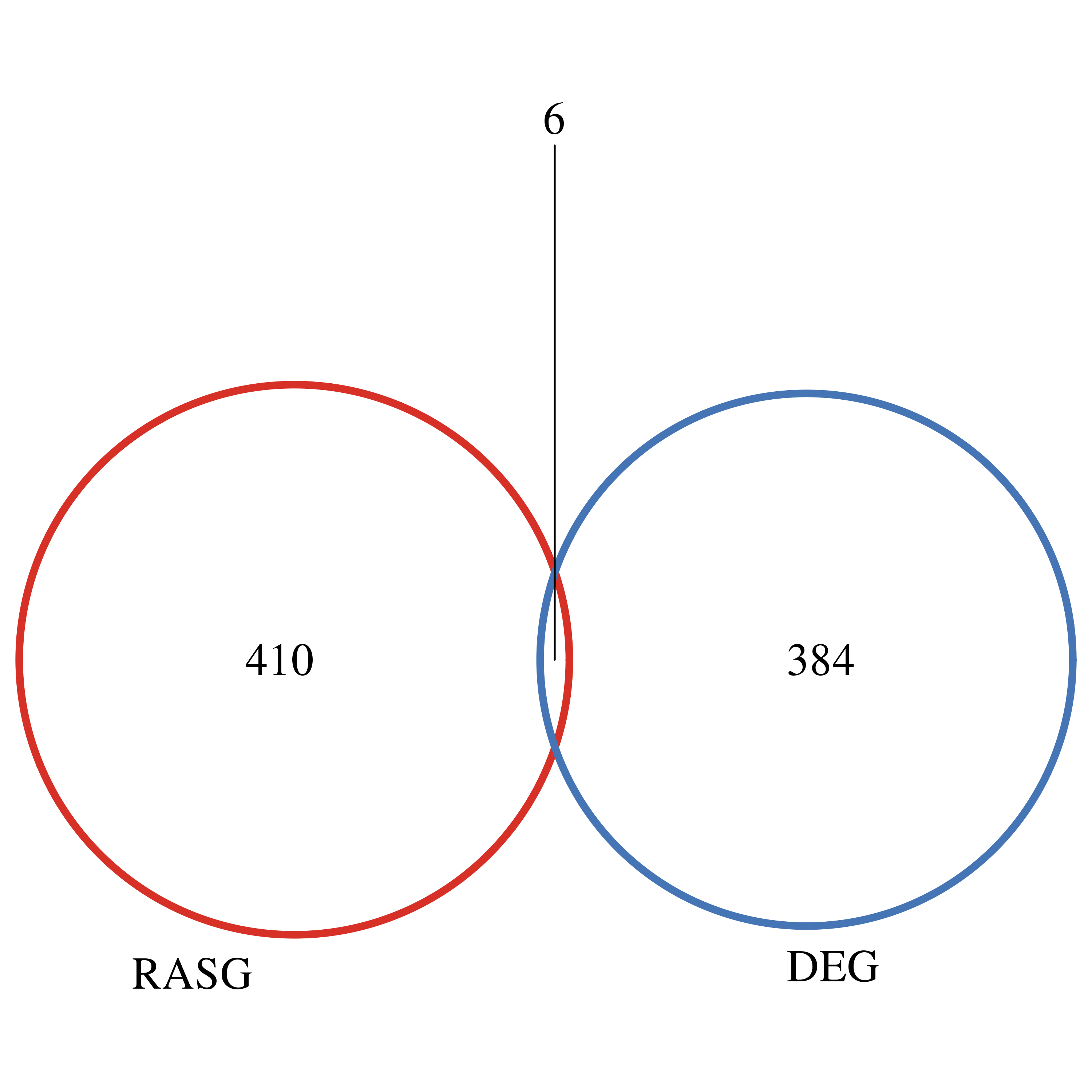

Supplement: FIGURE S2 [file OncolRes-31-30166-s002.tif]
